# Bilingual effects on cognitive control: a comprehensive bibliometric analysis of 50 years of research

**DOI:** 10.3389/fpsyg.2026.1905413

**Published:** 2026-08-06

**Authors:** Adam John Privitera, Jingxin Han, Kangyue Han, Jia'en Yee, Yuan Yin

**Affiliations:** 1 Advanced Institute of Humanities and Social Sciences, University of Electronic Science and Technology of China, Chengdu, China; 2 Science of Learning in Education Centre, National Institute of Education, Nanyang Technological University, Singapore, Singapore; 3 Centre for Research and Development in Learning, Nanyang Technological University, Singapore, Singapore; 4 Nanyang Technological University, Singapore, Singapore; 5 Linguistics and English Language, School of Social Sciences, Lancaster University, Lancaster, United Kingdom

**Keywords:** bilingual effects, bilingualism, cognitive control, executive function, science mapping

## Abstract

Claims of cognitive advantages associated with bilingualism have generated substantial research interest and controversy, particularly regarding influences on cognitive control. While multiple reviews have synthesized findings across previous work, their limited scope, focused exclusively on monolingual-bilingual comparisons, has precluded a more comprehensive understanding of the field as a whole. To address this, the present study provides a comprehensive bibliometric analysis of all published and unpublished original research investigating bilingual effects on cognitive control. Searches conducted in Web of Science and ProQuest Dissertations and Theses returned 8,266 documents of which 764 met eligibility criteria and were included in the analyses. Performance analysis, science mapping, and co-citation analysis were conducted using the bibliometrix package in R. Investigation of bilingual effects on cognitive control has grown steadily since the mid-2000s and is approaching saturation. Research output and influence are heavily concentrated among Western institutions, with binary monolingual–bilingual comparisons remaining the dominant methodological paradigm. Co-citation analysis identified three foundational thematic clusters reflecting an intellectual trajectory from early claims of universal bilingual advantages, through empirical reconsideration, toward more nuanced, experience-sensitive theoretical frameworks. These findings highlight persistent structural and methodological limitations in the field and underscore the need for greater global representation, improved measurement practices, and theoretically refined frameworks to advance understanding of how bilingualism shapes cognitive control.

## Introduction

Historically framed as a disadvantage (Darcy, 1953; Saer, 1923), bilingualism is now widely considered a desirable trait given the diverse range of linguistic, social, and cultural benefits it provides (Diamond, 2010). Beyond these well-established benefits, research emerging in the mid-twentieth century began to identify additional advantages associated with the use of multiple languages, commonly referred to as the “bilingual advantage” or more broadly conceptualized as “bilingual effects” (Barac and Bialystok, 2012; Privitera et al., 2023a). In this context, “bilingual effects” refers to a field-specific term used to describe reported differences or associations between bilingual experience and cognitive control outcomes, rather than to imply definitive causal relationships. Although initial studies challenging the prevailing deficit view reported superior nonverbal intelligence in bilinguals relative to monolinguals (e.g., Peal and Lambert, 1962), the majority of this work has focused on measures of cognitive control (Bialystok et al., 2022), attentional functions that are essential in controlling thought and behavior (Friedman and Miyake, 2017; Miyake et al., 2000). The cognitive demands of bilingualism, which include managing simultaneously active languages (Abutalebi and Green, 2007), are thought to lead to measurable improvements in cognitive control through adaptation of the underlying attention system (Bialystok, 2024; Bialystok and Craik, 2022). In contrast with the linguistic, social, and cultural benefits of bilingualism, claims of bilingual effects on cognitive control are met with considerable skepticism (Antoniou, 2019; Paap, 2019).

Much of the controversy surrounding research investigating bilingual effects on cognitive control stems from the mixed nature of findings. Earlier primary work questioning the veracity of previous findings and highlighting alternative explanations and methodological limits (Morton and Harper, 2007; Paap and Greenberg, 2013) led to stronger challenges from more recent systematic reviews and meta-analyses. Although positive accounts can be found (Grundy, 2020), published evidence syntheses have tended to report mixed (Van den Noort et al., 2019) or null associations between bilingualism and cognitive control (Lehtonen et al., 2018), with the magnitude of observed bilingual effects modulated by age and task characteristics (Ware et al., 2020), number of task trials (Hilchey and Klein, 2011), sample size (Lehtonen et al., 2018), and language pair (Carthery-Goulart et al., 2023). While these evidence syntheses have provided a deeper understanding of the influence of bilingualism on cognitive control, their exclusive focus on studies comparing monolingual and bilingual samples perpetuates an ecologically questionable view that monolinguals are an appropriate control against which bilinguals can be compared (Rothman et al., 2023). More importantly, the limited scope of these reviews has prevented the field from having a more comprehensive understanding of the size, scope, and characteristics of all research investigating bilingual effects on cognitive control.

Given the sustained interest and increasing research output in the investigation of bilingual effects on cognitive control (Privitera and Weekes, 2023; Sanchez-Azanza et al., 2017), much can be gained from a broadly-focused analysis of all existing work. While scoping and systematic reviews generally aim to investigate narrowly-focused research questions using a small number of studies (Arksey and O'Malley, 2005; Munn et al., 2018), bibliometric analyses are most appropriate for analyzing large amounts of scientific data with a broad investigative focus (Donthu et al., 2021). To date, one bibliometric analysis has examined this topic (Sanchez-Azanza et al., 2017), reporting increased publication output between 2005 and 2015 and a rise in number and impact of studies challenging positive bilingual effects on cognitive control after 2014. While this study provided valuable insights into the field, its focus on a relatively small number of studies published from 2005 to 2015 (*n* = 139) exclusively comparing monolingual and bilingual samples, coupled with the absence of comprehensive performance analyses and science mapping, has precluded a broader understanding of the evolution, structure, and intellectual development of the field. Unlike previous, narrowly-focused analyses, any new efforts should also consider the growing number of studies investigating these phenomena within samples of bilinguals, exploring how differences in language experience (i.e., the degree of bilingualism) influence cognitive control (e.g., Beniwal and Singh, 2026; Obenauf et al., 2025; Privitera et al., 2023a; Xie and Zhou, 2020). To that end, the conduct of an inclusive and exhaustive bibliometric analysis will identify comprehensive publication trends, the influence of specific journals, papers, and authors, patterns of collaboration, and foundational themes in the investigation of bilingual effects on cognitive control.

To provide an updated and comprehensive overview of the field, we conducted a bibliometric analysis of all published and unpublished original research investigating bilingual effects on cognitive control. This analysis provides detailed performance analysis metrics covering historic trends in publication, identifies the most prolific and influential contributors to the field, and highlights the most frequently used terms. We also map out collaboration networks at the country, institution, and researcher levels, and identify foundational themes in the field through co-citation analysis. Findings from the present study can provide researchers with insights into foundational themes, key players, and the most relevant platforms for the dissemination of work in this area. To the best of our knowledge, this is the first exhaustive bibliometric analysis of research investigating bilingual effects on cognitive control, addressing a critical gap in our understanding of the foundations of this highly significant and growing area of research.

## Methods

### Overall methodological approach

The present bibliometric analysis was conducted following guidance from Donthu et al. (2021). Bibliometric analysis is a quantitative research method that uses bibliographic metadata to systematically examine the structure and evolution of a scientific field (Broadus, 1987). Through analyses of publication characteristics, citation patterns, and collaboration networks, it identifies influential contributors, intellectual foundations, and emerging research trends. As opposed to scoping, systematic, and other reviews, bibliometric analyses are designed to characterize the scientific literature itself rather than synthesize the findings of individual studies (Grant and Booth, 2009). They focus on identifying patterns in research productivity, collaboration, and scholarly influence, thereby providing a macro-level perspective on the development and structure of a research field.

### Data collection

Data for the present study were identified through Web of Science (WoS) with searches conducted across both the Core Collection and ProQuest Dissertations and Theses Citation Index. The decision to use a single research platform was made to ensure citations downloaded adhered to a single format, preventing the need for manual formatting of results from other databases prior to analysis. While reliance on a single platform, albeit one with access to multiple databases, may not be appropriate during the conduct of a systematic review, this is considered best practice in bibliometric analysis (Chen, 2017; Donthu et al., 2021). The WoS platform is particularly appropriate given the quality of data available for bibliometric analysis and the large number of relevant journals indexed (Martín-Martín et al., 2021). Searches were initially performed on March 17th, 2025 with updated searches performed on February 9th, 2026. During both searches, fields were limited to article title, abstract, and keywords, with no date of publication limits applied. The finalized search string is presented in Table 1.

**Table 1:** Web of Science search string.

TI = (((bilingual* OR multilingual* OR dialect*) AND (inhibition OR switching OR shifting OR updating OR monitoring OR “working memory” OR “executive function*” OR “executive control” OR “attentional control” OR “cognitive control”) NOT (“natural language processing” OR therap* OR “speech recognition” OR “case study” OR “language teaching” OR impairment OR disorder OR patient OR aphasia OR review OR “meta-analysis”)))OR AB = (((bilingual* OR multilingual* OR dialect*) AND (inhibition OR switching OR shifting OR updating OR monitoring OR “working memory” OR “executive function*” OR “executive control” OR “attentional control” OR “cognitive control”) NOT (“natural language processing” OR therap* OR “speech recognition” OR “case study” OR “language teaching” OR impairment OR disorder OR patient OR aphasia)))OR AK = (((bilingual* OR multilingual* OR dialect*) AND (inhibition OR switching OR shifting OR updating OR monitoring OR “working memory” OR “executive function*” OR “executive control” OR “attentional control” OR “cognitive control”) NOT(“natural language processing” OR therap* OR “speech recognition” OR “case study” OR “language teaching” OR impairment OR disorder OR patient OR aphasia OR review OR “meta-analysis”)))

Additional Limits: Exact Search; Language: English; Format: Articles, Proceeding papers, Early access (applied to Core Collection search only). ProQuest Dissertation and Theses searches conducted through Web of Science were identical but omitted the author keyword (AK) field.

### Search and screening

Search results were exported in RIS format and uploaded into Covidence, an online review management platform. To ensure that only relevant documents were included during our analyses, search results were only included if they met the following eligibility criteria:

• Primary empirical studies;
• Article, thesis, dissertation, or conference proceedings
• Conducted in healthy participants;
• Focused on the impact of bilingualism or bilingual language experience on cognitive control;
• Assessed at least one domain of cognitive control (e.g., inhibition);
• Analyzed at least one index of cognitive control (e.g., Flanker task interference score) under at least one of the following conditions:
 o Compared bilingual and monolingual samples or subsamples;
 o Compared different bilingual samples or subsamples;
 o Tested the association between any measure of language experience (e.g., second language proficiency) and index of cognitive control within the same bilingual sample or subsample.
• Published or posted at any time;
• Written in English;

Evidence syntheses including systematic reviews and meta-analyses were excluded because their inclusion could alter citation counts, and collaboration and co-citation networks, obscuring patterns in the original research. To be as inclusive as possible, bilingualism was operationalized as the use of two or more languages or dialects, regardless of modality (e.g., sign language). All documents were screened by at least two independent reviewers at both the title/abstract and full text levels. At least two reviewers needed to agree before a given document was advanced to each subsequent stage of screening with disagreements settled through discussion with a third reviewer. Because the present study included both published and unpublished research, dissertations, theses, and conference papers selected for inclusion during the initial search were excluded if they were later published as journal articles, with only the published record included.

### Bibliometric analysis

Formal analyses were conducted in R (R Core Team, 2025) using the *bibliometrix* package (Aria and Cuccurullo, 2017), a free tool that supports both performance analysis and science mapping. After screening, bibliographic data for all documents selected for inclusion was exported from Covidence in CSV format for analysis using bibliometrix. After loading the bibliographic data file, keywords and WoS KeyWords Plus were automatically merged by bibliometrix. Data were then screened and revised to address issues with entries containing multiple names for an individual author (e.g., Bialystok E and Bialystok Ellen). Duplicate author names were first matched using institutional affiliations and were then revised to use the most commonly adopted version across all included documents. Next, the 100 most common keywords were screened and adjusted to prevent alternative spellings (e.g., working memory and working-memory) or synonyms (e.g., cognitive control and executive control) from being counted as unique items (word revision list available in Supplementary materials). Finally, affiliations were checked and modified to reflect specific institutions and not university systems (e.g., San Diego State University and not California State University System), and to remove redundant affiliations within a single citation that would inflate performance analysis measures. To reduce the potential for human error, author name and affiliation adjustments were performed using the find and replace function in Microsoft Excel and keyword adjustments were performed automatically in bibliometrix by uploading a spreadsheet of equivalent terms. Accuracy of author name, keyword, and affiliation revisions were confirmed through manually checking using tables generated in bibliometrix.

## Results

### Search, selection, and overview of dataset

**Figure 1:**
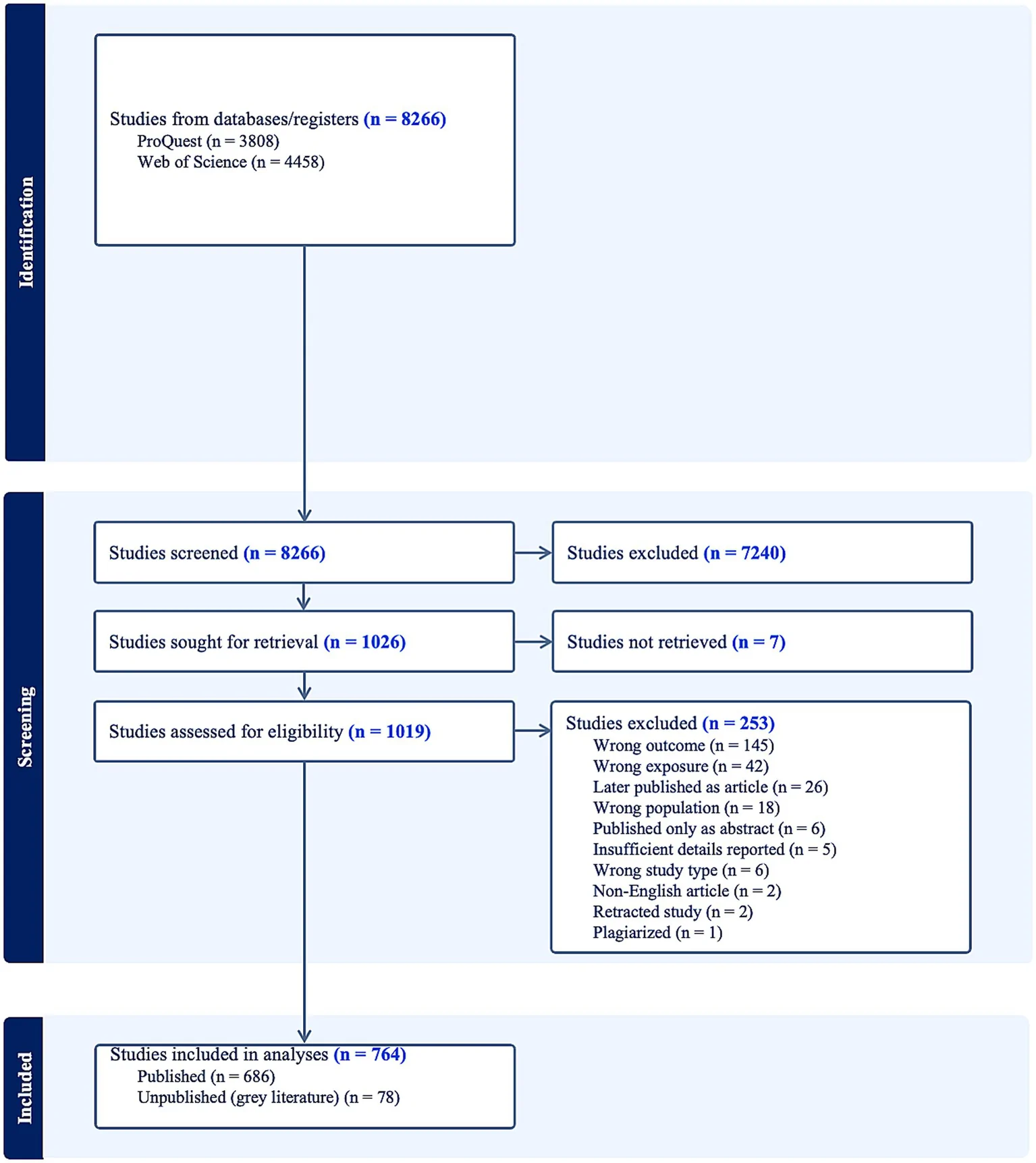
Search, screening, and selection overview.

An overview of the search, screening, and selection processes is summarized in Figure 1. Final searches across both databases returned a total of 8,266 documents for screening and selection. After the application of eligibility criteria, we were left with 764 unique documents. Details of the final analyzed dataset are reported in Table 2. Our dataset included 686 published articles and 78 unpublished theses, dissertations, and conference proceedings. Included documents were published or posted between 1976 and 2026 in a total of 154 different sources. This field is host to 1,590 individual authors with 1,199 of those authors (75%) contributing only a single document to the field. The vast majority of these documents (87%) were written by multiple authors, with an average of 3.35 co-authors per document. The high co-authorship rate, combined with international co-authorships accounting for nearly one-third of all research points to a highly interconnected and global research community in this area. Documents in our dataset received an average of 41.51 citations, indicating that, on average, research in this area has attracted significant attention and influence. Additionally, the vast number of references cited within the collection (24,409) highlights the deep scholarly foundation upon which this research is built, demonstrating that researchers in this area are engaging thoroughly with prior work and demonstrating a solid understanding of the existing literature.

**Table 2:** Dataset description.

Description	Results
Timespan	1976–2026
Sources	154
Documents	764
Annual growth rate	3.27%
Average citations per document	41.51
References	24,409
Keywords	1,086
Authors	1,590
Single-authored documents	103 (73 theses or dissertations); 93 authors
Co-authors per document	3.35
International co-authorship	30.76%

### Annual publication and citation analysis and life cycle

**Figure 2:**
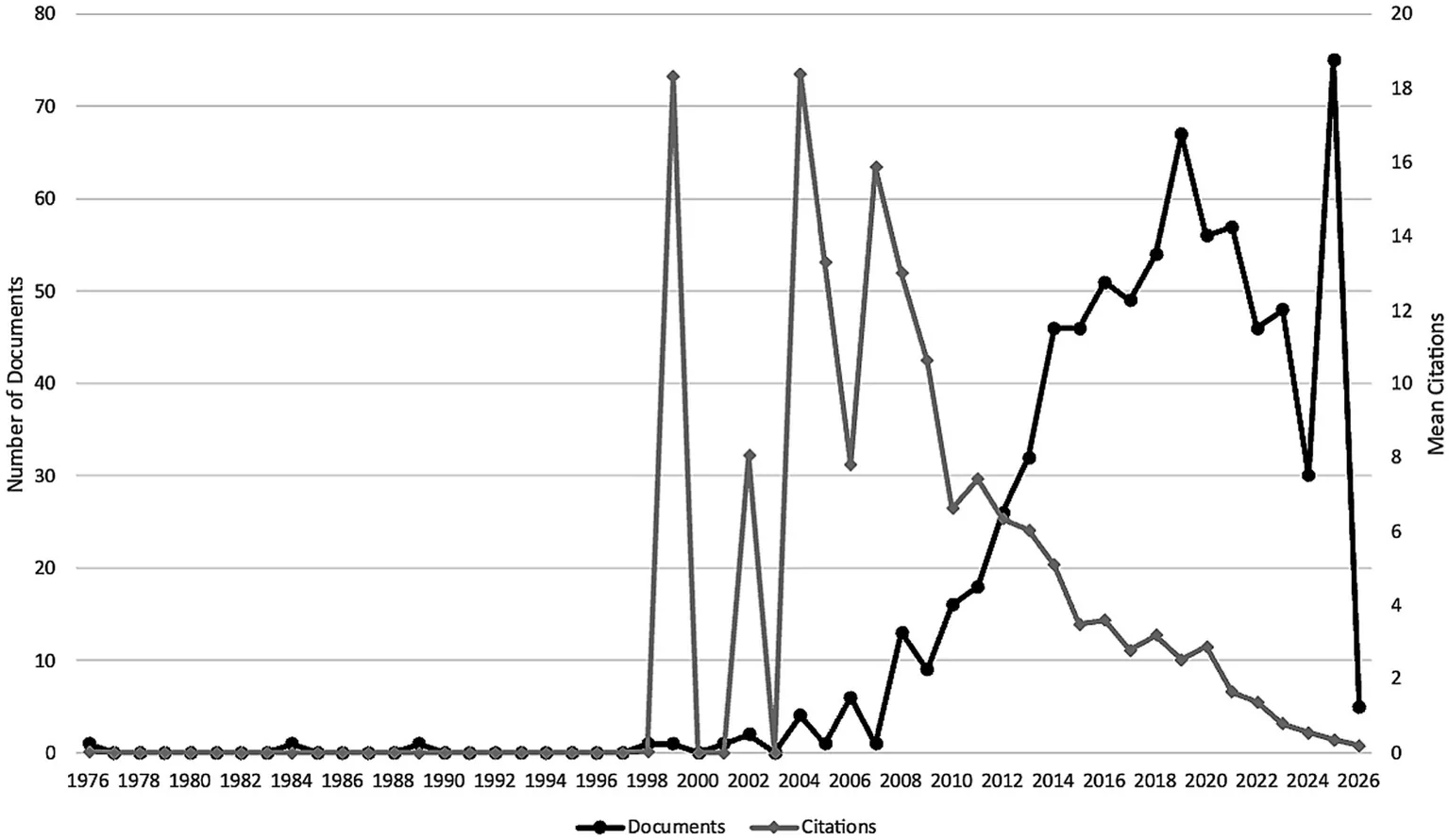
Publication and citation trends over time.

**Figure 3:**
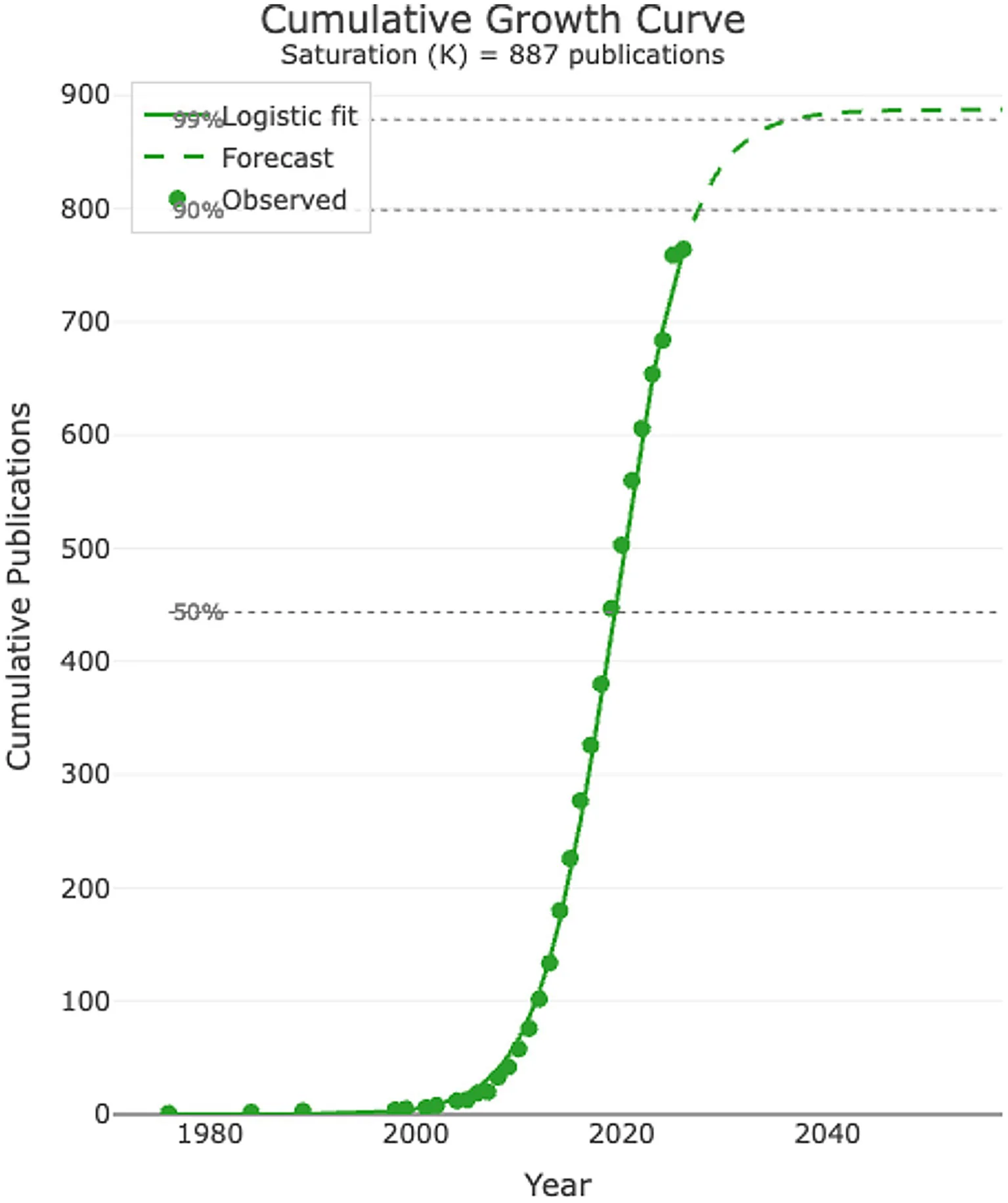
Life cycle of scientific production.

Publication and citation trends are presented in Figure 2, which includes both the total number of documents produced (black line, left y-axis) and the mean citations received by documents (grey line, right y-axis) for a given year. The left and right y-axes use different scales and should be interpreted independently, but together they illustrate the relationship between the growth of the literature and the scholarly influence of this work across different stages of the field’s development. Research investigating bilingual effects on cognitive control has shown an annual growth rate of 3.27%, suggesting a relatively stable trajectory characterized by steady output rather than rapid expansion or decline. However, visual inspection of publication trends (Figure 2) indicates that this stability did not emerge until around 2010. Research output first peaked in 2019 and, with the exception of 2021 and 2023, generally declined before reaching a second, higher peak in 2025. This overall pattern suggests that investigation of bilingual effects on cognitive control has entered a mature phase after the initial peak. Analysis of the scientific production lifecycle suggests that research concerning bilingual effects on cognitive control will likely be reaching 99% of its saturation level within the next 10 years (Figure 3).

The temporal pattern of publications and citations shown in Figure 2 suggests a clear shift in the development of research on bilingual effects on cognitive control. Early activity (pre-2008) was characterized by very low publication output but sporadic spikes in mean citations, likely reflecting the influence of a small number of highly cited foundational studies. From around 2009 onward, publication output increased markedly, indicating the rapid expansion of the field. This growth was accompanied by a steady decline in mean citations, which is consistent with both the recency of newer publications and the diffusion of citations across a larger body of work. In more recent years, output has remained relatively high despite fluctuations, while mean citations have continued to decrease, suggesting a transition toward a more mature and consolidated research area in which influence is distributed more evenly across studies rather than concentrated in a few seminal papers.

### Performance analysis

#### Most productive countries

The 10 most productive countries by research output are presented in Table 3. Researchers based in the United States, an officially monolingual country, produced the highest percentage of research, accounting for 21% of included documents. Another North American country, Canada, was also a top producer, accounting for 14% of included documents. While the majority of top research producing countries are from either North America or Europe, two countries from Asia, China and Singapore, are notable exceptions, accounting for 9 and 2% of produced research, respectively. Apart from the United States and Canada, no single country or territory accounted for more than 10% of research, suggesting that it is a topic of broad international interest. Levels of international collaboration, as measured by the number of documents with at least one co-author from a different country (multiple country publications; MCP), varied considerably across the most productive countries, averaging around 35% (Table 3). With the exception of Belgium, the two least internationally collaborative countries, the United States and Canada, were also the two most productive. This pattern suggests that researchers in these countries may rely more heavily on robust domestic collaboration networks. While these data may suggest that research in this area is led from a less internationally collaborative perspective, the observation that over 20% of publications from both the United States and Canada included at least one internationally affiliated co-author suggests this is not the case.

**Table 3:** Ten most productive countries.

Country	Documents	% of Total	TC	AVG Citations	% MCP
USA	161	21.07%	7,337	45.60	20.5%
Canada	106	13.87%	11,004	103.80	25.5%
UK	72	9.42%	1,804	26.10	37.5%
China	71	9.29%	760	10.70	36.6%
Spain	30	3.93%	2,812	93.70	36.7%
Belgium	29	3.80%	826	28.50	17.2%
The Netherlands	23	3.01%	1,161	50.50	39.1%
Italy	17	2.23%	839	49.40	52.9%
Singapore	17	2.23%	618	36.40	29.4%
Germany	15	1.96%	428	28.50	53.3%

Number of documents for each country was based on the country of the corresponding author’s affiliation. Total citations reflect the total number of citations received across sources indexed in Web of Science. AVG citations reflect the average number of total citations per document for a given country. TC, total citations; MCP, Multiple country publications.

Significant discrepancies were observed between the highest producing and most cited countries. While Canada was the second highest producer of research, work from this country was the most cited, receiving 1.5 times more citations than the United States, the highest producer. Work from researchers in Spain, the sixth most productive country, was also highly cited, receiving over twice as many average citations compared to work from the United States, and ranking third in total citations. Finally, although China was the fourth most prolific contributor to this area, it received the lowest number of average citations among the top 10 most productive countries. These notable differences between research output and research influence may reflect differences in research ecosystems (e.g., infrastructure, support, collaboration networks) or publication culture (e.g., prioritization of quality over quantity) across included countries.

#### Most productive institutions

The 10 most productive institutions are presented in Table 4. The United States, Canada, and the United Kingdom, were home to eight of the top 10 most productive institutions. These eight institutions accounted for 28% of all global research, suggesting that the core volume of the field is concentrated among a small group of leaders. Of note is the observation that three of the top 10 most productive institutions each accounted for over one-third of their respective countries’ total research output, suggesting that they serve as key hubs. This is most evident in the case of Vita-Salute San Raffaele University, which was responsible for 88% of all work in this area produced by researchers in Italy. In another notable case, York University in Canada accounted for 61% of all research produced by Canadian researchers and nearly 9% of all research worldwide. This latter finding suggests that York University is not only a key national hub, but serves as a lead international contributor to research investigating bilingual effects on cognitive control.

**Table 4:** Ten most productive institutions.

Institution	Country	Documents	% of Country	% of Total
York University	Canada	65	61.32%	8.51%
University of Toronto	Canada	29	27.36%	3.80%
University of London	UK	23	31.94%	3.01%
Baycrest Health Sciences	Canada	21	19.81%	2.75%
University of Edinburgh	UK	21	29.17%	2.75%
San Diego State University	USA	19	11.80%	2.49%
University College London	UK	18	25.00%	2.36%
Northwestern University	USA	17	10.56%	2.23%
Vita-Salute San Raffaele	Italy	15	88.24%	1.96%
Ghent University	Belgium	14	48.28%	1.83%

Total % across all institutions within a single country can exceed 100% due to dual-affiliations and collaborative research.

#### Researchers: productivity and influence

The 10 most productive researchers, arranged by research output, are presented in Table 5. Overall, the top 10 researchers, who represented less than 1% of all researchers in this field, authored 23% of all documents in the dataset. Top producing authors exhibited similar patterns of research output with one notable exception: Ellen Bialystok from York University in Canada. Bialystok, who is considered the pioneer in the investigation of bilingual effects on cognitive control, was the single most productive researcher, contributing 64 documents to the field starting in 1999. Studies which included Ellen Bialystok as an author accounted for 8% of all research in this area. By comparison, the second most productive researchers, Viorica Marian from Northwestern University in the United States and Antonella Sorace from the University of Edinburgh in the United Kingdom, both contributed less than one fourth this number (15 each). All other top producing researchers contributed between 11 to 13 documents to the dataset.

**Table 5:** Ten most productive researchers by output.

Author	Documents	*h*-index	*g*-index	LC	1st year pub
Bialystok, E.	64	44	64	3,013	1999
Marian, V.	15	13	15	229	2011
Sorace, A.	15	10	15	135	2009
Craik, F. I. M.	13	13	13	1,059	2004
Filippi, R.	13	9	12	80	2012
Abutalebi J.	12	7	12	119	2012
Luk, G.	12	10	12	594	2008
Xie, Z. L.	12	6	12	93	2014
Yang, H. J.	12	8	12	213	2011
Costa, A.	11	11	11	915	2008

Data reported in this table were calculated using only those articles returned from our search (i.e., local impact and influence) and should not be considered indices of any author’s global impact or influence. LC, local citations.

Initially mirroring the organization of authors by research output, Ellen Bialystok was the most influential researcher in the investigation of bilingual effects on cognitive control with a local citation count (3,013) nearly three times higher, and an h-index (44) over three times higher than the next most influential author, Fergus Craik from the Rotman Research Institute at Baycrest in Canada. The late Albert Costa (1970–2018) from the Universitat Pompeu Fabra in Spain, and Gigi Luk, initially at York University in Canada and later, Harvard University in the United States and McGill University in Canada, were the third and fourth most influential authors, collectively having over 1,500 local citations for their contributions beginning in 2008. Similar to the pattern observed across the most productive countries, there were a number of clear discrepancies between the most productive and most influential researchers, with only one researcher (Bialystok) given the same rank for both productivity and number of local citations.

The significant role played by Ellen Bialystok in the investigation of bilingual effects on cognitive control is worth noting. Although she was included as an author on 64 separate documents, Bialystok is the sole author on only four, suggesting regular collaboration. Of the top 10 most productive authors, two have published extensively with Bialystok. In one case, Bialystok was a contributor on all documents that included Fergus Craik as an author. In the other, Bialystok was a contributor on 10/12 (83%) documents that included Gigi Luk as an author. Both Fergus Craik and Gigi Luk collaborate regularly with Ellen Bialystok, with the latter having previously completed her PhD under Bialystok’s supervision. Of the articles which included Bialystok and Craik or Luk, four included all three authors. Collectively, the collaborations between Bialystok, Craik, and Luk account for 19/64 (30%) of Bialystok’s total contributions to this field and 2% of all research investigating bilingual effects on cognitive control.

#### Journals: output and influence

Supplementary Table 1 presents the 10 journals that have published the greatest number of studies on bilingual effects on cognitive control. The journal *Bilingualism: Language and Cognition* from Cambridge University Press published the largest number of papers in the field, accounting for 15% of all research, with papers receiving the highest number of total citations. Although only publishing 20 articles in this area, *Cognition* from Elsevier was the second most influential journal as measured by total citations, with published documents receiving an average of 156 citations. This notable discrepancy between research output and research influence can potentially be attributed to *Cognition’s* broader focus, which may have prioritized the publication of articles with wider appeal in this area of research. A similar pattern can be seen with other more broadly-focused journals including *Developmental Science* (140 average document citations) and *Journal of Experimental Child Psychology* (65 average document citations). Of the 10 journals with the highest number of publications, the largest number were published by Elsevier (40%) followed by Taylor & Francis (20%).

#### Most influential documents

Supplementary Table 2 presents the most widely cited documents in the present dataset, all of which were journal articles. Of note is the observation that total citations in Web of Science indexed sources were 2–3 times higher than local citation counts for the top 10 most influential articles, suggesting that these contributions were relevant in other areas of research. Of the 10 most influential articles in this area, six included at least one of the 10 most influential researchers. Only four of the most influential researchers (Bialystok, Craik, Luk, and Costa) contributed to these articles and only one (Costa) was outside the collaboration network of Ellen Bialystok. Nearly all of the most influential papers (7/10) were published in the top 10 most productive journals in this area, suggesting a preference for submitting findings to specialized journals or those broadly read within the behavioral sciences.

The most influential article as measured by both local and total citations is the article “Bilingualism, Aging, and Cognitive Control: Evidence From the Simon Task,” by Bialystok et al. (2004) published in the journal *Psychology and Aging.* This article is noteworthy for being the first report of bilingual effects on cognitive control in an adult sample, building on earlier work described in Bialystok’s (1992) seminal chapter in the book *Cognitive Processing in Bilinguals*. Ellen Bialystok is also an author on three additional articles in the top 10 (Bialystok et al., 2008; Bialystok and Martin, 2004; Martin-Rhee and Bialystok, 2008). Similar to her most influential article, these focus on identifying differences in cognitive control performance between monolingual and bilingual samples, but across three distinct age groups: children, younger adults, and older adults. The methodological approach adopted in these articles, specifically the comparison of monolingual and bilingual groups on cognitive control task performance, has had a lasting impact on the trajectory of research in this area, with its influence still clearly evident in contemporary work (Privitera et al., 2026).

Interestingly, Martin-Rhee and Bialystok’s (2008) study in children shares a common thread with the third (Costa et al., 2008), fourth (Costa et al., 2009), fifth (Carlson and Meltzoff, 2008), and seventh (Prior and MacWhinney, 2010) most influential articles. Across all five studies, the authors began to delineate the boundary conditions of previously reported bilingual effects on cognitive control. In particular, the importance of employing tasks with high cognitive control demands became evident, as differences between monolinguals and bilinguals were generally observed only under these conditions, especially when participants were near their cognitive peak (Costa et al., 2009). In addition, these studies helped clarify how such effects manifest, with converging evidence shifting focus away from an exclusive focus on measures of inhibition and toward measures of attentional control, which now underpin more recent theoretical accounts (Bialystok and Craik, 2022).

The second most influential article, Paap and Greenberg’s (2013) article published in *Cognitive Psychology* titled, “There is no coherent evidence for a bilingual advantage in executive processing,” represents a key moment in the field where the veracity of bilingual effects on cognitive control was called into question. Across a series of three studies in young adults, the authors not only failed to replicate previously reported bilingual effects, but actually identified evidence supporting significantly worse inhibition in bilinguals relative to monolinguals on the Simon task. Additional evidence for a “bilingual disadvantage” in inhibition was identified through regression analyses in which higher levels of bilingualism, operationalized as the summed proficiency across a participant’s languages, were associated with larger flanker and Simon effects even after accounting for influential variables including nonverbal intelligence. While Paap and Greenberg’s (2013) article was the most influential account against previously reported bilingual effects on cognitive control, an earlier study by Morton and Harper (2007) had already begun to explore alternative explanations for these effects. In their paper, “What did Simon say? Revisiting the bilingual advantage,” published in *Developmental Science*, the authors identified no support for superior cognitive control in bilingual children when compared with monolinguals from comparable ethnic and socioeconomic backgrounds, variables that had previously been ignored. These two influential studies contributed significantly to discussions around the need to improve methodological rigor in the investigation of bilingual effects on cognitive control.

#### Most frequent terms

Keywords were missing from seven included journal articles and all (73) dissertations and theses. Cleaning and adjustment of keywords resulted in the replacement of 29 alternative keywords under 13 different standard keywords including use of “cognitive control” in place of the terms “executive control” and “executive function” and “advantage” in place of “advantage” “bilingual advantage” and “bilingual advantages.” a spreadsheet containing all replaced keywords can be viewed on Open Science framework (https://osf.io/4efu8/overview?view_only=37cc466c4abd47c1aa037a7583822f58).

The most frequently used terms present in keywords, document titles, and abstracts are presented in Table 6, revealing both a conceptual core and differences in emphasis across sections. In the keyword field, *cognitive control* (832) and *bilingual* (445) dominate, alongside terms such as *advantage*, *inhibition*, and *working memory*, indicating that the field is strongly anchored in the investigation of cognitive control and, in particular, the debated “bilingual advantage.” In titles, *bilingual* (639) remains most prominent, with *control* (229) and *executive* (228) also commonly used, suggesting that authors tend to focus on the population of interest while still signaling the cognitive constructs under investigation. By contrast, abstracts exhibit a broader and more differentiated vocabulary, with *bilingual* (3,571), *control* (1,207), and *monolingual* (1,181) leading, alongside terms such as *children*, *advantage*, and *language proficiency*, likely reflecting more detailed reporting of study characteristics.

**Table 6:** Ten most frequently used terms across different fields.

Keywords	Freq	Titles	Freq	Abstracts	Freq
Cognitive control	832	Bilingual	639	Bilingual	3,571
Bilingual	445	Control	229	Control	1,207
Advantage	365	Executive	228	Monolingual	1,181
Inhibition	295	Children	126	Executive	863
Working memory	227	Advantage	123	Children	683
Children	148	Monolingual	114	Advantage	674
Language proficiency	136	Memory	111	Memory	500
Attention	128	Language proficiency	60	Differences	406
Interference	120	Switching	52	Language proficiency	397
Individual differences	114	Adults	51	Switching	373

General words that would not indicate the conceptual or methodological focus of a paper, such as “task” or “language” were not counted in the tabulation of respective word frequencies.

Taken together, these patterns support several inferences about the structure of the literature. First, the consistent prominence of *bilingual* and *cognitive control* across all sections indicates a high degree of conceptual cohesion, suggesting that the field is tightly organized around a core research question. Second, the prominence of terms such as *inhibition*, *working memory*, and *attention* in keywords but not titles or abstracts suggests that these may represent specialized components of cognitive control that are not consistently foregrounded, reflecting variability in construct operationalization across studies (Friedman and Miyake, 2017). Third, the strong presence of *monolingual* in abstracts and titles highlights the fundamentally comparative nature of this research, where contrasts between bilingual and monolingual groups are central, despite growing criticism (Rothman et al., 2023). Finally, frequent reference to different age groups and subject variables (e.g., *individual differences*, *language proficiency*) suggests an increasing sensitivity to more nuanced accounts aimed at identifying the conditions under which these effects emerge and which factors modulate them (e.g., Dash et al., 2022; Gullifer et al., 2021; Titone and Tiv, 2023; Xie et al., 2024).

### Science mapping

#### Country collaboration network

**Figure 4:**
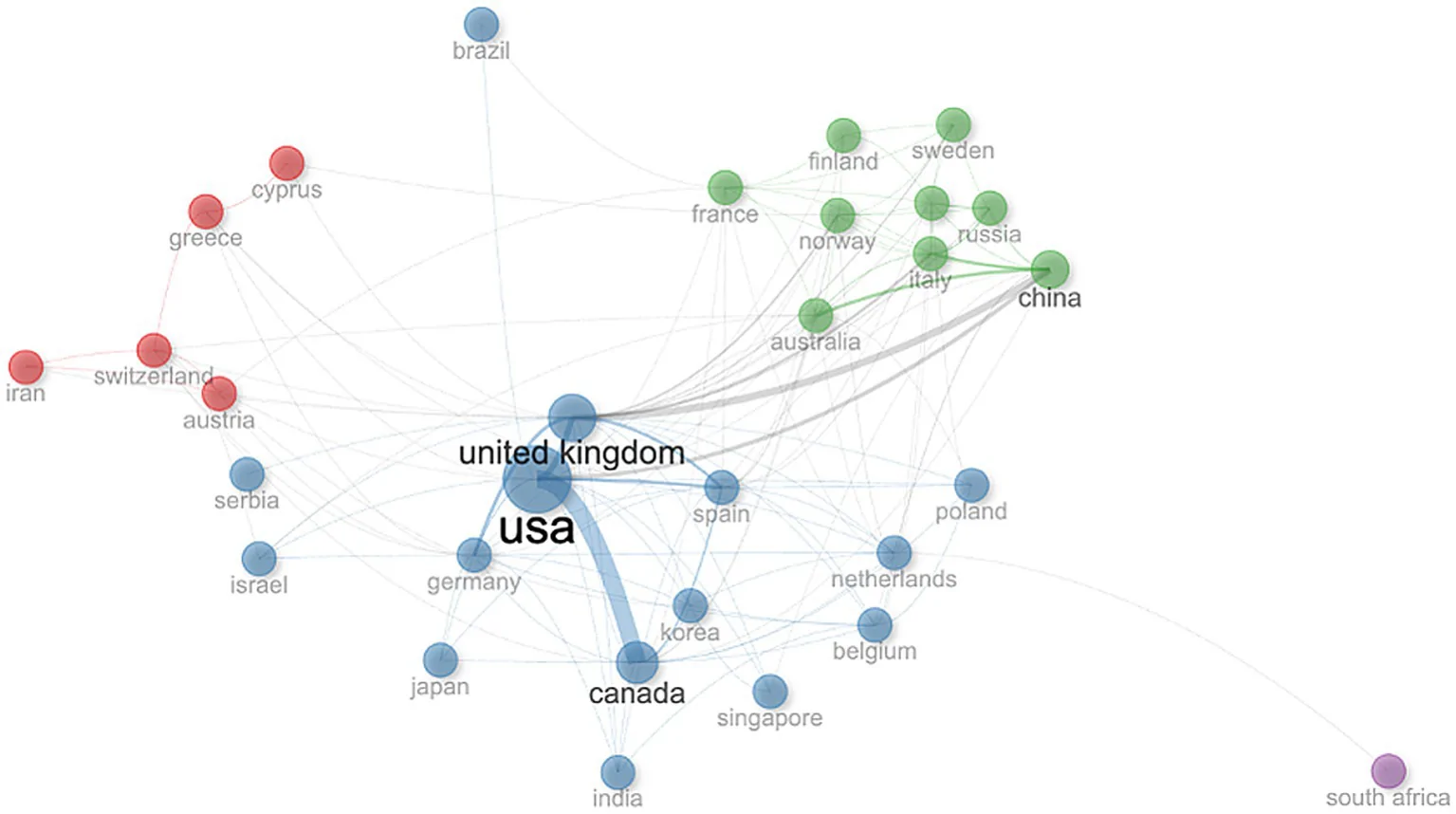
Country collaboration network (separate colors correspond to different clusters).

The country collaboration network (Figure 4), constructed using a Fruchterman–Reingold layout, Walktrap community detection algorithm, and association strength normalization, reveals a moderately dense and structured global research landscape. Node size reflects overall collaboration activity, while edge thickness captures the strength of partnerships. The network is organized around two dominant hubs, the United States and the United Kingdom, whose large node sizes and multiple strong connections indicate their central roles as primary drivers of international collaboration. Countries are integrated into the network through direct or indirect connections, with substantial variation in connectivity, as some nations serve as key hubs while others occupy peripheral or bridging positions. For example, Australia appears to link otherwise distinct clusters, while France connects more loosely at the margins of its community. In contrast, South Africa is nearly isolated, suggesting very limited collaboration with the other countries in this network.

Community detection identified several distinct clusters of collaboration. The largest (blue) community comprises a globally diverse group including the United States, United Kingdom, Germany, Canada, and several European and Asian countries, reflecting a highly established research network. A second (green) community, centered around China, includes a mix of European and Eurasian countries (e.g., Italy, Russia, and the Nordic states) alongside Australia, indicating more geographically concentrated but well-integrated collaboration. A smaller (red) community, consisting of countries such as Iran, Switzerland, Austria, Greece, and Cyprus, appears even more regionally concentrated and may reflect specialized research partnerships. These communities highlight both the cohesion of intra-group collaborations and the broader structure of global scientific cooperation.

At the node level, the United States and United Kingdom form the backbone of global collaboration, with extensive connections to countries such as Canada and Germany. China represents another major hub, particularly within its own community, with strong ties to the United Kingdom, United States, and Australia. Other countries, including Germany, Canada, France, Italy, and Australia, contribute to network cohesion through intermediary roles that connect regional and cross-community collaborations.

Overall, the network reflects a multi-polar system characterized by the dominance of English-speaking countries alongside the rapid growth of China as a distinct center of collaboration. While communities demonstrate strong internal cohesion, the presence of bridging connections suggests that knowledge exchange is not entirely compartmentalized.

#### Institution collaboration network

**Figure 5:**
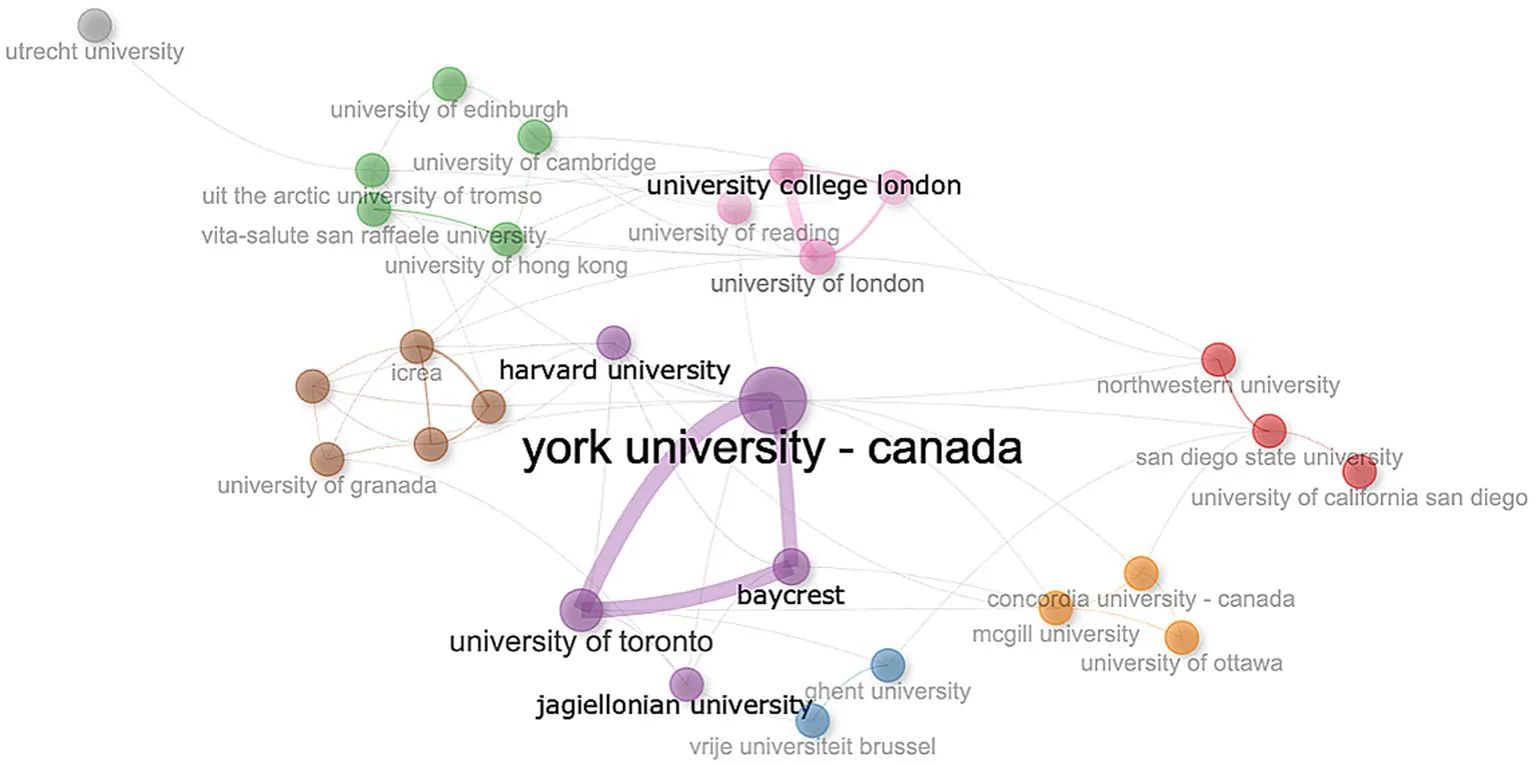
Institutional collaboration network (separate colors correspond to different clusters).

The institution collaboration network (Figure 5), constructed using a Fruchterman–Reingold layout, Walktrap community detection algorithm, and association strength normalization, moderately dense and well-structured pattern of partnerships, characterized by both clustering and centralization. Nodes (limited to a maximum of 30 to improve readability; isolated nodes removed) represent institutions, with size indicating the extent of collaborative activity, while edges denote co-authorship ties. The network exhibits a clear hub-and-spoke configuration, with a central core linking multiple surrounding clusters. Most institutions are connected either directly or indirectly to this central region, suggesting a broadly integrated collaboration landscape, although some nodes (e.g., Utrecht University) appear more peripheral, indicating more limited engagement within the dataset.

Community detection identified several distinct clusters that reflect underlying collaboration patterns, shaped by geography, institutional alliances, and shared research interests. The dominant central community (purple) is anchored by York University, the University of Toronto, and Baycrest Health Sciences in Canada, forming the primary nexus of connection and collaboration. This central community also hosts Jagiellonian University in Poland, a crucial node connecting the dominant community with smaller, more regionally-focused communities in Europe. Surrounding this core are several smaller but cohesive clusters, including a United Kingdom and Europe-oriented group (green) with a distinct connection to the University of Hong Kong, a United Kingdom-centric cluster (pink), and two distinct but connected North American clusters focused on the United States (red) and Canada (orange). It is worth noting that the University of Hong Kong serves as the only non-Western institution in this network, sharing a connection with Vita-Salute San Raffaele University. This connection is through Jubin Abutalebi, a highly prolific and influential researcher who earned his PhD from the University of Hong Kong and is a faculty member at Vita-Salute San Raffaele University. Additional country-focused communities include a cluster of Spanish institutions (e.g., ICREA and University of Granada), and a Belgian cluster (Vrije Universiteit Brussel and Ghent University). Despite their distinct identities, these communities remain interconnected through links to the central hub, facilitating cross-cluster collaboration.

At the institution level, York University emerges as the most prominent and central node in the network, reflecting its high level of collaborative activity across multiple communities. This central position is closely associated with the contributions of Ellen Bialystok, the most productive and influential author in the field, whose extensive collaborations have connected York University with institutions such as the Rotman Research Institute at Baycrest, Harvard University, and McGill University. Other institutions hosting highly productive and influential researchers, including the University of Edinburgh (Antonella Sorace), University of London (Roberto Filippi), and Northwestern University (Viorica Marian), also demonstrated substantial collaborative activity within their respective clusters. Overall, the network reflects an interconnected but hierarchically structured system in which a small number of institutions serve as central hubs supporting knowledge exchange and international collaboration.

#### Researcher collaboration network

**Figure 6:**
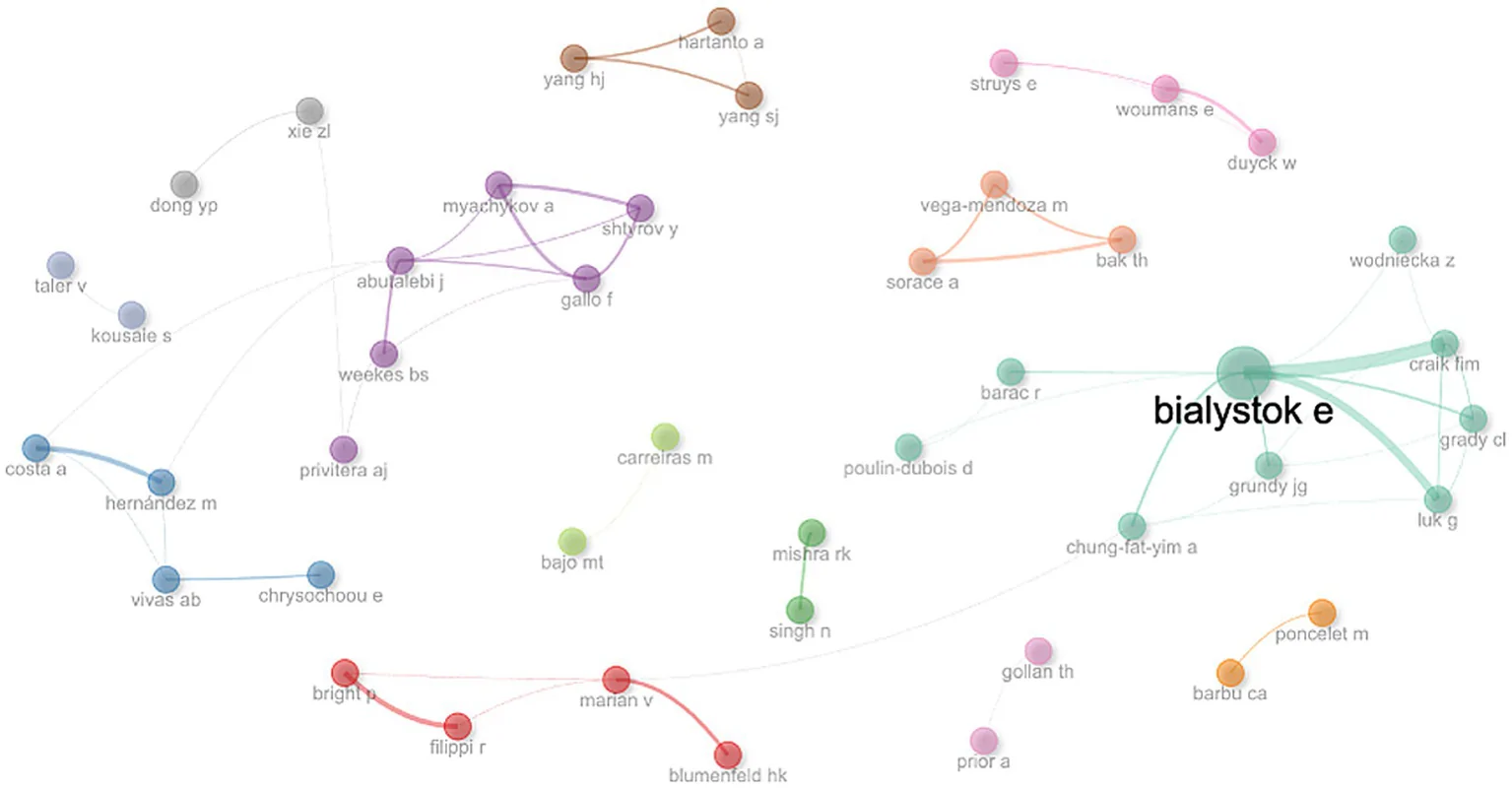
Researcher collaboration network (separate colors correspond to different clusters).

The researcher collaboration network is presented in Figure 6. The same parameters as those used to generate the institution collaboration network were used in the generation of this network. Nodes (limited to a maximum of 50 to improve readability; isolated nodes removed) represent the top researchers as measured by the number of collaborations, and edge thickness between nodes reflect the strength of those collaborations. This collaboration network reveals a largely fragmented and sparse structure, characterized by multiple distinct clusters rather than a single, highly interconnected component. Although most authors are connected within localized groups, the network overall lacks strong global integration, indicating that collaboration is typically confined to smaller geographically-defined groups.

The most prominent community (green) is centered on Ellen Bialystok, forming the largest and most cohesive group in the network. This cluster includes a core set of closely connected collaborators (e.g., Fergus Craik, Gigi Luk, and John Grundy), alongside more peripheral contributors who are thematically aligned but less directly integrated. Additional communities, such as those comprising authors like Federico Gallo and Jubin Abutalebi (purple), Albert Costa and Mireia Hernández (blue), and Viorica Marian and Roberto Filippi (red), represent smaller, internally cohesive groups that function as independent collaboration units. Numerous dyads and triads further highlight the presence of highly localized or specialized partnerships, reinforcing the segmented nature of the field.

At the individual level, Ellen Bialystok emerges as the dominant hub, exhibiting the highest productivity and strongest collaborative ties. Her central position reflects her substantial contribution to the field and extensive integration within the largest collaboration network. Other authors function as local hubs within their respective clusters, although none demonstrate comparable levels of connectivity. Despite limited bridging connections between communities, several researchers occupy important intermediary positions. For example, Ashley Chung-Fat-Yim connects the dominant cluster with the cluster containing Viorica Marian and Roberto Filippi, while Adam John Privitera links researchers in Hong Kong and Mainland China, including Xie Zhilong, one of the most productive researchers in the field.

Overall, the network reflects a decentralized yet unevenly structured collaboration landscape, in which a single highly influential hub coexists with multiple smaller, relatively independent communities. These findings point to a field that is both specialized and segmented, with collaboration concentrated within established groups rather than distributed across the broader scholarly network.

#### Co-citation network analysis

**Figure 7:**
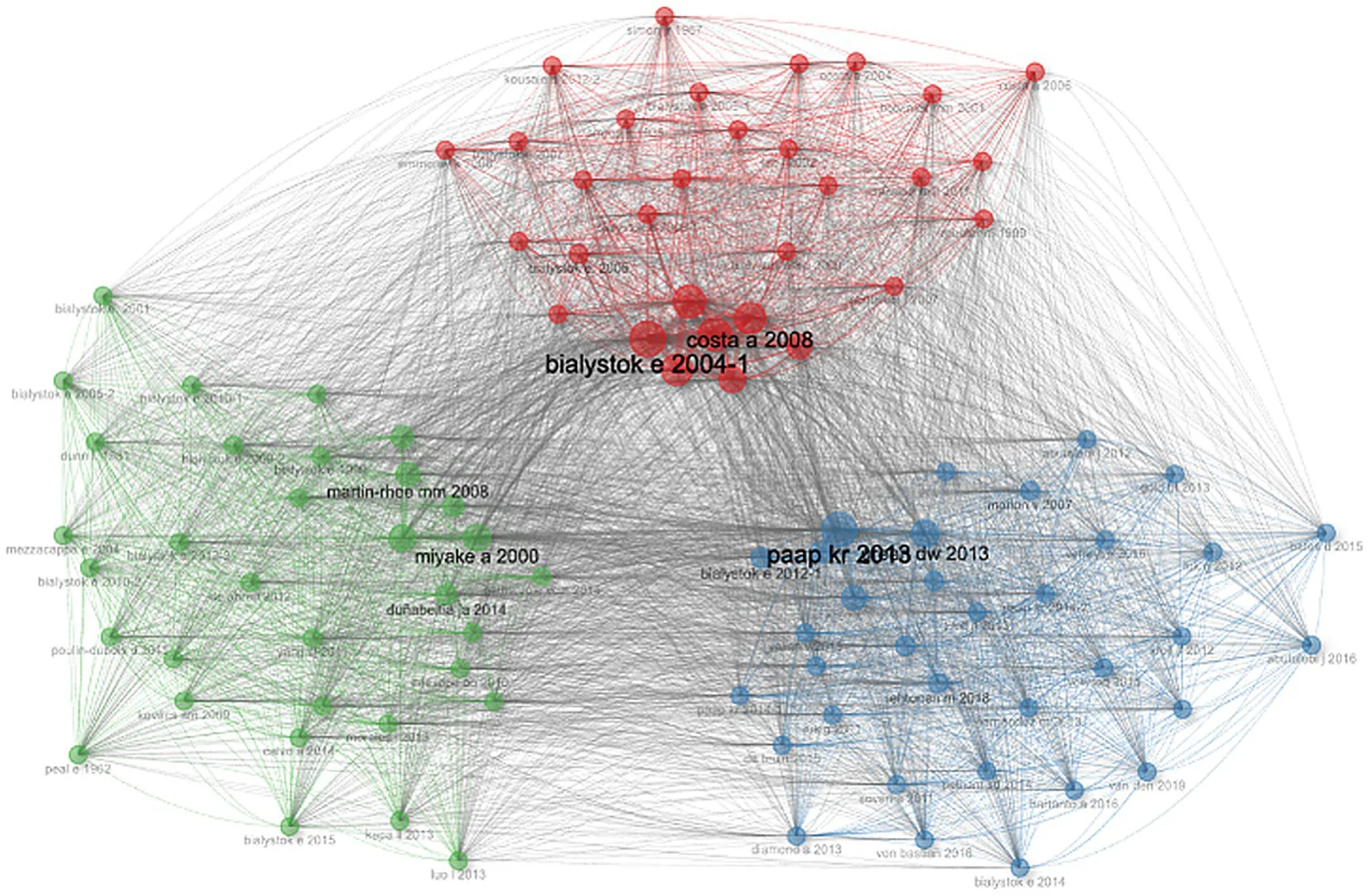
Co-citation network (separate colors correspond to different clusters).

We used co-citation analysis to explore the foundational themes in this area by analyzing reference lists from published studies (Donthu et al., 2021). A co-citation is created when papers are cited together in the reference list of another paper. We constructed a network of co-citations using 24,409 references cited across 764 documents to identify the foundational themes in the investigation of bilingual effects on cognitive control. This network was generated using a Fruchterman–Reingold layout and Louvain community detection algorithm with total node number limited to 100 and nodes with less than two edges or isolated nodes representing papers with no co-citations removed. Network analysis resulted in the emergence of three clusters representing foundational themes in the field (Figure 7). Summaries of the representative papers are provided below.

#### Red cluster: bilingual effects in adults and theoretical foundations

This cluster is anchored by the earliest report of bilingual effects on cognitive control in an adult sample (Bialystok et al., 2004), which exhibits the highest betweenness centrality in the network, indicating its critical role as a bridge between different research areas. Green's (1998) theoretical account, serves as another major hub. In this paper, Green describes the inhibitory control model which argues that bilinguals manage language competition by suppressing the non-target language, and that this experience trains their inhibition, resulting in a cognitive advantage over monolinguals, particularly in cognitive control tasks. Two papers from Albert Costa and colleagues describe empirical investigations of cognitive control in young adult bilingual populations. This includes early work extending these investigations into the domain of attentional control (Costa et al., 2008) as well as additional work identifying border conditions of these phenomena (Costa et al., 2009). Finally, a review from Hilchey and Klein (2011) provided some of the earliest evidence in support of more general bilingual effects on cognitive control, extending beyond the prevailing focus on inhibition. Their synthesis of previous studies investigating nonlinguistic interference tasks (e.g., Simon) identified more consistent bilingual effects on monitoring, evidenced in improved performance on both congruent and incongruent task trials. In contrast, little evidence supported the robustness of bilingual effects on inhibition, making a case for bilingual executive processing advantage as opposed to the originally proposed bilingual inhibitory control advantage. While a later review by Hilchey et al. (2015) reported that their initial support for a bilingual executive processing advantage did not stand the test of time, it should be acknowledged that neither review was exhaustive in their inclusion of research in this area. Additional notable works in this cluster (e.g., Fan et al., 2002; Stroop, 1935) show connections to classic attention and cognitive control paradigms which are still widely used. Collectively, the articles in this cluster establish the theoretical and empirical foundations of bilingual effects on cognitive control, and the high betweenness centrality of these articles suggests they serve as conceptual bridges connecting bilingual research with broader cognitive psychology literature.

#### Blue cluster: empirical reconsiderations and theoretical refinements

The second cluster centers on Paap and Greenberg’s highly influential article (Paap and Greenberg, 2013) which provides a through empirical critique of the reliability and generalizability of bilingual effects on cognitive control. A second similar contribution from Paap et al. (2015) strengthened the argument made in their earlier paper, synthesizing null and mixed findings across a range of behavioral and neuroimaging studies. The presence of a meta-analysis by Lehtonen et al. (2018) in this cluster, reporting null bilingual effects across a range of cognitive control measures after correcting for publication bias, further underscores its focus on critically reevaluating the foundational claims established in the first cluster.

While not directly challenging earlier accounts, three additional articles provided important theoretical updates on the relationship between bilingualism and cognitive control. Building on Green's (1998) inhibitory control model, the adaptive control hypothesis (Green and Abutalebi, 2013) underscores the significance of the interactional context in modulating the cognitive demands of bilingualism. This account proposes three language contexts experienced by bilinguals: *single-language* (e.g., Mandarin at home and French at work), *dual-language* (e.g., English with teachers and Tamil with friends at school), and *dense code-switching* (e.g., freely switching between Spanish and English within an utterance). Of these, dual-language contexts are proposed to impose the greatest cognitive demands and may be most associated with bilingual effects on cognitive control.

Similarly, Bialystok et al.’s and colleagues review articles build on earlier frameworks such as Green’s (1998) inhibitory control model by retaining the assumption that bilingual language production requires regulation of competing linguistic representations. However, while Green’s (1998) model emphasizes inhibition of the non-target language, Bialystok et al. and colleagues extend this account by embedding language control within broader domain-general systems. Specifically, the earlier review (Bialystok et al., 2012) links language regulation to processes such as monitoring and switching, with a focus on adults and cognitive reserve (Stern, 2002), where the later (Bialystok, 2017) adopts a broader lifespan perspective, emphasizing executive attention and experience-dependent plasticity. These two articles reframed bilingual effects as dynamic adaptations that extend beyond inhibition alone, a position aligning with the most update theoretical accounts (Bialystok, 2024; Bialystok and Craik, 2022). Collectively, the papers in this cluster reflect a critical reevaluation and theoretical refinement of earlier claims, emphasizing the inconsistency of reported bilingual effects while advancing more nuanced, context-dependent and mechanistic accounts grounded in adaptive control and experience-driven plasticity.

#### Green cluster: early accounts of nonlinguistic bilingual effects in children

This cluster emphasizes developmental aspects of the relationship between bilingualism and cognitive control with a focus on children. This group of studies is unified by a shared cognitive control framework, most prominently grounded in Miyake et al. (2000), which conceptualizes cognitive control as comprising separable but related components including inhibition, shifting, and updating. Aligning with this framework, subsequent investigations replicated and extended early findings of bilingual effects in children, localizing these effects primarily to processes of inhibition and conflict resolution. Using the Dimension Change Card Sort (DCCS; Zelazo et al., 1996), Bialystok and Martin (2004) reported that bilingual preschool children outperformed their monolingual peers on measures of *conflict resolution and interference suppression*. Across a battery of tasks including the DCCS, the Attention Network Test (ANT; Rueda et al., 2004), and delay of gratification (Mischel et al., 1989), Carlson and Meltzoff (2008) found that bilingual kindergarteners demonstrated superior performance on tasks involving conflict and interference relative to monolingual peers, but showed no advantage on response inhibition. In a similar vein, Martin-Rhee and Bialystok (2008) provided further evidence suggesting that bilingualism selectively enhances interference suppression rather than response inhibition in children.

Of the studies in this cluster with the highest betweenness centrality, Morton and Harper (2007) stands out as one of the earliest reports of an alternative explanation for previously reported findings: unmeasured differences in socioeconomic status (SES). In a sample of young children, the authors identified that, while monolinguals and bilinguals did not differ in their Simon task performance, higher SES children outperformed their lower SES peers. Methodologically, these studies converge on developmental, experimental designs, typically focusing on preschool and early school-aged children and employing standardized behavioral paradigms such as conflict and rule-switching measures. This approach reflects an emphasis on isolating separable components of domain-general cognitive control rather than language-specific skills. Empirically, the studies overlap in their identification of bilingual effects in conflict monitoring and interference control rather than in more basic or global processes, suggesting that reported effects are selective and task-dependent. Collectively, this cluster represents foundational work that frames bilingualism as enhancing specific components of cognitive control within a developmental context, establishing the theoretical and methodological basis for subsequent research and, eventually, debate in the field.

## Discussion

This study presents an exhaustive, methodologically rigorous bibliometric analysis on research investigating bilingual effects on cognitive control. Through the analysis of a bibliometric dataset spanning 50 years, we report historic publication trends, productivity and influence metrics, patterns of collaboration, and foundational themes in this significant area of research. This bibliometric analysis provides a comprehensive overview of the field that is not presently addressed by existing research syntheses, including more focused systematic reviews and meta-analyses. Findings from the present review can provide valuable insights to researchers interested in better understanding or advancing our understanding of the relationship between bilingualism and cognitive control.

### Geographic concentration of research output and influence

Performance analysis highlighted a Western-centric concentration of research investigating bilingual effects on cognitive control. Of the top 10 most productive countries (Table 3), eight were located in Western contexts, with two North American countries, the United States and Canada, serving as the highest producing and most influential countries, respectively. While both China and Singapore are among the top 10 most prolific contributors to research in this area, their total output accounts for around 12% of all research, compared with the nearly 60% of research contributed by the most productive Western countries. A similar pattern can be seen across the most productive institutions (Table 4), and researchers, (Table 5), with zero non-Western institutions and only two non-Western researchers included. The concentration of research suggests that theoretical and empirical accounts of bilingual effects are disproportionately influenced by Western research agendas and norms, highlighting a gap in the knowledge base of the field.

Western-centric dominance can also be observed in the most influential papers (Supplementary Table 2), which are all produced by authors from Western institutions. Consequently, the most dominant accounts may reflect culturally bound perspectives on language experience and cognition, over representing bilingual effects that emerge in Western language contexts. This noted dominance of research from Western, educated, industrialized, rich, democratic (WEIRD) countries mirrors a similar bias in the broader behavioral and cognitive sciences (Henrich et al., 2010), a factor that can limit generalizability to bilinguals worldwide. Despite the Western bias present in performance metrics, the percentage of multiple country publications across top producing countries (Table 3) as well as the country collaboration network (Figure 4) support that the influence of such bias might be partly addressed through international collaboration.

### Theoretical and methodological evolution

Research on bilingual effects on cognitive control has undergone a fundamental shift, moving from early claims of universal cognitive advantages to more sophisticated, context-dependent theoretical frameworks. Performance analysis of the most influential papers (Supplementary Table 2), and visual inspection of the co-citation network (Figure 7) indicates that, while seminal studies documenting bilingual effects on cognitive control (e.g., Bialystok et al., 2004; Carlson and Meltzoff, 2008) retain significant influence, they now exist within a more complex theoretical landscape that includes substantial critical evaluation and methodological refinement. The prominence of methodological critiques (Paap and Greenberg, 2013; Paap et al., 2015), as well as evidence syntheses reporting mixed or null findings (De Bruin et al., 2015; Lehtonen et al., 2018), suggests that the field has progressed beyond simple confirmation or refutation toward more nuanced accounts of these phenomena. This evolution reflects a transition from early exploratory work on the general cognitive consequences of bilingualism to more recent research aimed at identifying the specific conditions under which bilingual effects emerge, and the factors that modulate their magnitude.

Early theoretical frameworks, particularly Green's (1998) inhibitory control model, provided foundational hypotheses about domain-general cognitive enhancement conferred through bilingual experience. Although several influential early behavioral studies offered support for this account (e.g., Bialystok et al., 2004; Carlson and Meltzoff, 2008; Martin-Rhee and Bialystok, 2008), subsequent findings demonstrating bilingual effects in domains beyond inhibition (e.g., Prior and MacWhinney, 2010) prompted a reevaluation of this framework. Syntheses of the growing body of evidence led to revised accounts of how bilingual experience may shape cognitive control more broadly, extending beyond inhibition-specific predictions (Bialystok, 2017; Bialystok et al., 2012). Contemporary frameworks exemplified by the adaptive control hypothesis (Green and Abutalebi, 2013) represent a theoretical advancement, reconceptualizing bilingualism as a multifaceted experience with context-dependent cognitive consequences. This shift toward experience-based models, further evidenced in keyword trends (Table 6), suggests that the field is developing more refined theoretical frameworks capable of accounting for the heterogeneity observed across influential evidence syntheses.

Comparing performance between monolingual and bilingual samples has been the most widely adopted method for investigating bilingual effects. While this approach has been instrumental in the earliest seminal work, including the most influential papers in this area identified using both performance analysis (Supplementary Table 2) and co-citation analysis (Figure 7), continued adoption of this methodology perpetuates the false assumption that monolinguals are a suitable baseline against which to compare bilinguals (Rothman et al., 2023). From a global perspective, this assumption is increasingly difficult to justify, as bilingualism represents the most common linguistic profile worldwide (De Houwer, 2021). The widespread use of monolingual control groups therefore not only frames bilingualism as a deviation from the norm, but also introduces potential confounds that may obscure the identification of genuine bilingual effects including differences in cultural background, educational experience, and patterns of language use. This approach reduces language status to an oversimplified and largely uninformative categorical label, obscuring substantial variability within both bilingual (De Bruin, 2019; DeLuca et al., 2019; Gullifer et al., 2021; Luk and Bialystok, 2013) and monolingual populations (Castro et al., 2022). Although recent research has begun to adopt more nuanced approaches by examining variability within bilingual samples (e.g., Grundy et al., 2020; Privitera et al., 2022), the prevalence of the term “monolingual” in titles and abstracts (Table 6) suggests that reliance on traditional monolingual-bilingual comparisons remains substantial.

### Implications and future directions

Given the current Western-centric focus of research investigating bilingual effects on cognitive control, there is a need for future research to increase the diversity of populations investigated. Institutions and governments can support such efforts by establishing research agendas that support international collaboration efforts. Given the current network of international collaboration (Figure 4), and multiple country publication rates among the nations with the highest research output (Table 3), there is already a strong foundation that can be leveraged. Current collaboration networks support that efforts are needed to build stronger bridges between leading research groups and collaborators in underrepresented areas, particularly with institutions in Global South countries. These efforts will help diversify the linguistic representation in this area of inquiry, helping to address notable representation issues across broader areas of behavioral research (Henrich et al., 2010). To maximize the impact of these and other collaborations, researchers should consider alignment with best practices in Open Science including the open sharing of data (Bolibaugh et al., 2021).

Publication and life cycle trends (Figures 2, 3) suggest that research investigating bilingual effects on cognitive control has entered a mature phase after initial peaks in research output over the last decade. This period should serve as a time of continued methodological and theoretical refinement, continuing to address influential critiques (Paap and Greenberg, 2013), mixed or null findings identified by evidence syntheses (De Bruin et al., 2015; Lehtonen et al., 2018), and methodological trends that undermine the strength of conclusions and generalizability of findings (Luk and Bialystok, 2013; Privitera et al., 2023b; Privitera and Weekes, 2023; Rothman et al., 2023). Better accounting for the range of potentially confounding variables highlighted by influential methodological critiques identified in our co-citation analysis (Figure 7) including culture (Samuel et al., 2018), SES (Morton and Harper, 2007), and other individual differences (Van den Noort et al., 2019) can help isolate the true relationship between bilingualism and cognitive control. Continued innovation in the analysis of data, including the wider adoption of statistical methods that best account for the multi-trial data structure of behavioral tasks, such as mixed effects models (Bates et al., 2015; Linck and Cunnings, 2015), or nonlinear effects, such as generalized additive models (Miwa and Baayen, 2021) can help further address the limits present in the extant literature (Veríssimo, 2021). Reconsidering commonplace analysis methods is especially crucial given evidence that reliance on conventional methods like linear regression can result in the identification of spurious bilingual effects (Privitera et al., 2023b).

Alternative ways of measuring cognitive control that are reliable and valid should also be explored. Given the prominence of classic behavioral tasks evidenced in our co-citation analysis (Figure 7), and the observation that many of these tasks suffer from low reliability, only weakly correlating with each other (Hedge et al., 2018; Paap and Sawi, 2016), there is a need to refine how our construct of interest in measured. Recently, Burgoyne et al. (2023) proposed a series of “squared” versions of the classic Stoop, Flanker, and Simon tasks that have been widely used in the study of bilingual effects. These freely available tasks (https://osf.io/7q598/) have demonstrated high levels of internal consistency, test–retest reliability, and correlate with other established measures of cognitive control. To the best of our knowledge, these tasks have not been used in any investigation of bilingual effects. Beyond tasks, there is also value in reconsidering which measures from these tasks are most useful in our work. Adopting atypical measures of task performance is extremely rare, with only a few previous studies analyzing indices generated from diffusion modeling (Ong et al., 2017), ex- Gaussian analysis (Abutalebi et al., 2015), and nonlinear modeling of time-sensitive performance trajectory (Privitera, 2026). Importantly, mixed findings that permeate this field may, in part, stem from the near-exclusive reliance on conventional statistical tests and averaged performance measures, highlighting a critical need to reevaluate which methods best support the identification of genuine bilingual effects on cognitive control.

### Limitations

Findings from this bibliometric analysis should be considered in light of some limitations. The dataset used across all analyses was sourced exclusively from WoS. While searches were extended to include theses, dissertations, and conference proceedings, our results are based on only resources that are indexed by WoS, which tend to overrepresent STEM journals. Reliance on a single database is generally considered best practice when conducting a bibliometric analysis due to inter-database differences in metadata organization and quality (Chen, 2017; Donthu et al., 2021). Documents written in any non-English language were also excluded, which further reduced the exhaustiveness of our search. However, the impact of this methodological decision is likely very limited given that the vast majority of research worldwide is published in English (e.g., Ramírez-Castañeda, 2020). Finally, clusters identified during co-citation analysis are influenced by temporal bias, more strongly capturing historically influential research as opposed to more recent work. Accordingly, these clusters should be interpreted as a representing the intellectual structure of the field and not the current research frontier (Jarneving, 2005).

## Conclusion

The present study maps 50 years of research investigating bilingual effects on cognitive control, revealing a field that has matured from early foundational claims of universal cognitive advantages into a sophisticated, actively contested area of inquiry. Performance analysis identified a persistent Western-centric concentration of research output and influence, with binary monolingual–bilingual comparisons remaining the dominant, and widely criticized, methodological paradigm. Co-citation analysis maps out an intellectual trajectory from seminal empirical work establishing bilingual effects on inhibition and conflict monitoring, through influential critical challenges, toward more nuanced, experience-sensitive theoretical frameworks. As the field approaches saturation, there exists an important opportunity for further theoretical and methodological refinement, greater global representation, and the adoption of approaches that best capture the multidimensional heterogeneity inherent in bilingual experience. Together, these efforts will support the building of a comprehensive and valid understanding of how bilingualism shapes cognitive control.
