# Supplementary material for: Bilingual effects on cognitive control: a comprehensive bibliometric analysis of 50 years of research
Source: Front Psychol. 2026 Aug 6;17:1905413. doi: 10.3389/fpsyg.2026.1905413 (PMC13490175; doi:10.3389/fpsyg.2026.1905413)
Supplement: Supplementary file 1 [file Table_1.docx]

**Supplementary Table 1**. Top 10 journals by output

| **Source** | **Publisher** | **Articles** | **TC** | **AVG Citations** |
| --- | --- | --- | --- | --- |
| *Bilingualism* | Cambridge | 117 | 3,657 | 31.26 |
| *Frontiers in Psy* | Frontiers Media | 64 | 2,160 | 33.75 |
| *Int. J. Biling* | Sage | 35 | 549 | 15.69 |
| *Int. J. Biling. Educ. Biling* | Taylor & Francis | 23 | 468 | 20.35 |
| *J Cog Psy* | Taylor & Francis | 22 | 953 | 43.32 |
| *J. Exp. Child Psy* | Elsevier | 22 | 1,434 | 65.18 |
| *Cognition* | Elsevier | 20 | 3,113 | 155.65 |
| *Neuropsychologia* | Elsevier | 16 | 814 | 50.88 |
| *Dev. Science* | Wiley | 15 | 2,093 | 139.53 |
| *J. Neuroling* | Elsevier | 15 | 387 | 25.80 |

Note: Total citations reflect the total number of citations received for all documents published in a given source across sources indexed in Web of Science. AVG citations reflect the average number of total citations per document for a given source. TC: total citations

**Supplementary Table 2**. 10 most influential papers

| **Paper** | **DOI** | **LC** | **TC** |
| --- | --- | --- | --- |
| Bialystok, E. (2004) *Psy. Aging* | 10.1037/0882-7974.19.2.290 | 328 | 1,049 |
| Paap, K.R. (2013) *Cog. Psy* | 10.1016/j.cogpsych.2012.12.002 | 301 | 738 |
| Costa, A. (2008) *Cognition* | 10.1016/j.cognition.2006.12.013 | 265 | 701 |
| Costa, A. (2009) *Cognition* | 10.1016/j.cognition.2009.08.001 | 263 | 567 |
| Carlson, S.M. (2008) *Dev. Science* | 10.1111/j.1467-7687.2008.00675.x | 226 | 678 |
| Bialystok, E. (2008) *J. Exp. Psy. Learn* | 10.1037/0278-7393.34.4.859 | 212 | 629 |
| Prior, A. (2010) *Bilingualism* | 10.1017/S1366728909990526 | 184 | 394 |
| Martin-Rhee, M.M. (2008) *Bilingualism* | 10.1017/S1366728907003227 | 180 | 412 |
| Bialystok, E. (2004) *Dev. Science* | 10.1111/j.1467-7687.2004.00351.x | 177 | 554 |
| Morton, J.B. (2007) *Dev. Science* | 10.1111/j.1467-7687.2007.00623.x | 151 | 317 |

Note: Data are arranged based on highest number of local citations which was calculated using only those articles returned from our search. Total citations reflect the total number of citations received for all documents published in a given source across sources indexed in Web of Science. LC: Local citations; TC: Total citations
